# PTPN2 in the Immunity and Tumor Immunotherapy: A Concise Review

**DOI:** 10.3390/ijms231710025

**Published:** 2022-09-02

**Authors:** Jiachun Song, Jinxin Lan, Jiaping Tang, Na Luo

**Affiliations:** Department of Anatomy and Histology, School of Medicine, Nankai University, Tianjin 300071, China

**Keywords:** PTPN2, inflammation, tumor immunotherapy

## Abstract

PTPN2 (protein tyrosine phosphatase non-receptor 2), also called TCPTP (T cell protein tyrosine phosphatase), is a member of the PTP family signaling proteins. Phosphotyrosine-based signaling of this non-transmembrane protein is essential for regulating cell growth, development, differentiation, survival, and migration. In particular, PTPN2 received researchers’ attention when Manguso et al. identified PTPN2 as a cancer immunotherapy target using in vivo CRISPR library screening. In this review, we attempt to summarize the important functions of PTPN2 in terms of its structural and functional properties, inflammatory reactions, immunomodulatory properties, and tumor immunity. PTPN2 exerts synergistic anti-inflammatory effects in various inflammatory cells and regulates the developmental differentiation of immune cells. The diversity of PTPN2 effects in different types of tumors makes it a potential target for tumor immunotherapy.

## 1. Introduction

The counterbalance of protein tyrosine phosphatases (PTPs) and protein tyrosine kinases (PTKs) controls the protein tyrosine phosphorylation level, which plays a critical role in cellular signaling [1]. The human genome encodes 107 PTPs, which are divided into four families according to the amino acid sequence of the catalytic domain (Table 1) [2]. PTPN2 belongs to the intracellular non-receptor PTP subgroup, the classical PTP subfamily, the largest class I cysteine PTP family [3].

The PTPN2 gene (protein tyrosine phosphatase non-receptor 2) encodes for the PTPN2 protein also called TCPCP (T cell protein tyrosine phosphatase), originally cloned from a human T cell cDNA library. It is a member of the PTP family of signaling proteins that are thought to play a role in cell growth, differentiation, the mitotic cycle, and oncogenic transformation. In humans, PTPN2 exists as two functional variants (45 kD and 48 kD) due to alternative splicing. The larger PTPN2 (48 kD) variant resides in the endoplasmic reticulum because of a hydrophobic C-terminus masking the nuclear localization sequence. The smaller PTPN2 (45 kD) variant is primarily in the nucleus due to bipartite nuclear localization.

As a signaling molecule, PTPN2 regulates a variety of cellular processes by dephosphorylating either receptor protein tyrosine kinases, such as EGFR (epidermal growth factor receptor) [5], CSF1R (Colony stimulating factor 1 receptor) [6], PDGFR (Platelet-derived growth factor receptor) [7], IR (insulin receptor) [8] or non-receptor protein tyrosine kinases, such as JAK (janus family kinases) [9], Src (src family kinases), or STAT (signal transducers and activators of transcription) family kinases [10], either in the cytoplasm or nucleus.

PTPN2 is associated with pathological processes, including inflammatory responses, immune disorders, and tumor development. Recently, PTPN2 has gained tremendous interest, primarily due to 2 findings. One is genome-wide association studies (GWASs), which revealed that loss-of-function single-nucleotide polymorphisms (SNP) in the PTPN2 gene confer a predisposition for the onset of inflammatory bowel disease and the development of immune disorders (e.g., Crohn’s disease, Type 1 diabetes, rheumatoid arthritis, and celiac disease) [11,12]. The other is in vivo genetic screening utilizing a CRISPR-Cas9 delivery system that identifies Ptpn2 as a cancer immunotherapy target. Deletion of PTPN2 in tumor cells increased IFN-γ signaling and antigen presentation to T cells, along with amplified growth arrest in response to cytokines, suggesting its therapeutic potential in potentiating immunotherapy efficacy [13]. 

Here, we reviewed recent research concerning the role and impact of PTPN2 on inflammatory/immune responses and tumor therapy to achieve better application of PTPN2.

## 2. The Structure of PTPN2

The PTPN2 gene maps to chromosome 18p11.3-p11.2 [14]. This 100 kb gene comprises 10 exons, with exons 1–7 encoding a conservative PTP catalytic domain [15]. In rat cells, 4 alternative splice variants of PTPN2 were discovered: PTP-S1, PTP-S2, PTP-S3, and PTP-S4, with PTP-S2 (TC45) and PTP-S4 (TC48) being the most common. In human cells, only two splice variants were found (TC45 and TC48) due to the loss of the internal splice site by mutation [16]. 

**Figure 1:**
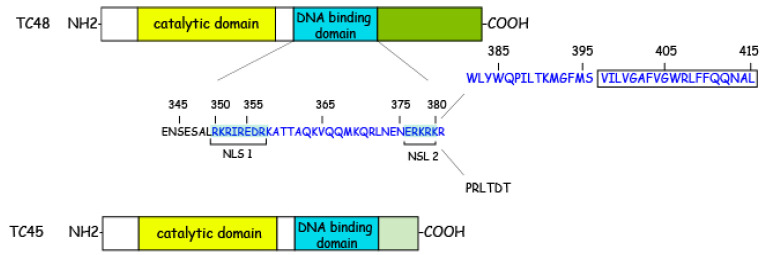
Structure of 45 kD and 48 kD PTPN2 variants. This image indicates the catalytic and non-catalytic C-terminal domains of PTPN2. Its catalytic domain highlights the high degree of primary sequence conservation. The DNA binding domain contains two basic clusters (residues 350–358 (RKRIREDRK) and 377–381 (RKRKR)) that form a bipartite nuclear localization signal (NLS). TC45 localizes to the nucleus by virtue of NLS. The hydrophobic C-terminal tail of TC48 must override the bipartite NLS to permit the targeting of the ER. The region shown in blue (from 350–415) is where sites in TC48 interact with p23 and p25.

These two isoforms of PTPN2 share the same catalytic domain consisting of 272 amino acids but differ at their C-terminus [17,18]. The larger TC48 variant, comprising 415 amino acids, possesses a hydrophobic C-terminus, which contains 66 amino acids (from 350–415) that are necessary for binding of p23 and p25 for the sake of targeting PTPN2 to the endoplasmic reticulum [19,20]. The smaller TC45 variant, consisting of 387 amino acids, has a specific bipartite nuclear localization sequence in the C-terminal region that targets it to the nucleus and binds to DNA (Figure 1).

There is controversy about the order of the appearance of the two isoforms. One theory is that alternative splicing dramatically alters the character of the C-terminus by removing the hydrophobic tail (VILVGAFVGWRLFFQQNAL) of TC48 to generate TC45 [18]. The other theory suggests that TC45 is generated by a unique exon at the C-terminus, which codes for 6 hydrophilic amino acids (PRLTDT). TC48 is generated by the penultimate exon and is an extension of the 3′ intron. This results in the replacement of C-terminal 6 amino acids of TC45 with 34 amino acids (WLYWQPILTKMGFMS VILVGAFVGWRLFFQQNAL) in TC48 [16].

These two isoforms differ in many ways. First, they localize at different sub-cellular compartments: Although it lacks a traditional ER retention motif, the stretch of 19 hydrophobic residues at the extreme C-terminus of TC48 masks the NLS, which targets it to the ER [16], though studies have shown that it can also be localized to the nuclear membrane [17]. In contrast, TC45 localizes to the nucleus due to NLS. Second, they have different substrate specificity and modulatory mechanisms. Together with their tightly regulated localization, this supports the idea that each isoform has a different cellular function [21].

### 2.1. Anti-Inflammatory Role of PTPN2

Abnormal expression of PTPN2 results in the occurrence of many inflammatory diseases. The anti-inflammatory role of PTPN2 is highlighted by the fact that PTPN2-deficient mice die a few weeks after birth because of systemic inflammation and severe colitis [22]. Loss of functional variants in PTPN2 is associated with an increased risk of developing chronic inflammatory disorders [12].

### 2.2. PTPN2 in Intestinal Inflammation

The PTPN2 gene has gained clinical interest recently since many SNPs in the 18p11 locus are associated with chronic inflammatory diseases [23]. More specifically, recent studies have demonstrated that the rs2542151 SNP is associated with inflammatory bowel disease, Crohn’s disease, and ulcerative colitis [24]. At the RNA and protein levels, PTPN2 mRNA and protein expression levels are elevated within the epithelial cells in inflammatory bowel disease (IBD). In addition, IBD-related inflammatory cytokines (e.g., IFN-γ, TNF) increase PTPN2 expression in an intestinal epithelial cell (IEC) line [25]. The deficiency of PTPN2 exacerbates barrier dysfunction after IFN-γ treatment [26]. Thus, IBD-inflammatory cytokines demonstrate a negative feedback loop whereby the loop triggers the expression of its own negative regulator of the signaling pathway [25].

PTPN2 influences intestinal inflammation by regulating (1) intestinal barrier function, (2) inflammatory factors, and/or (3) associated inflammatory immune cells.

The lack of PTPN2 expression in IECs significantly impacts the formation of an effective intestinal barrier. PTPN2 knockdown in IECs exacerbates the barrier disorders caused by IFN-γ treatment [26]. In addition, PTPN2 knockdown in IECs increases the expression of cation-selective pore-forming proteins, allowing the paracellular passage of cations into the intestinal lumen, which leads to intestinal fluid loss [26,27]. In addition to the damaged intestinal barrier, the secretion and related signaling mechanisms of inflammatory factors regulated by PTPN2 also affect the inflammatory response in the intestine. PTPN2 deficiency leads to an IFN-γ-mediated barrier defect in chronic intestinal inflammatory diseases associated with STAT1 signaling [26,28]. It also enhances the inhibitory effect of epidermal growth factor on intestinal epithelial chloride secretion [29] and promotes TNFα-induced secretion of cytokines [30]. Additionally, the loss of PTPN2 is associated with TNFα-induced extracellular signal-regulated kinase 1/2 (ERK1/2) and p38, without affecting c-Jun N-terminal kinase (JNK) or NF-κB phosphorylation signaling. In addition, the loss of PTPN2 potentiates TNFα-induced secretion of interleukin 6 (IL-6) and IL-8. These data indicate that PTPN2 activity plays a crucial role in the establishment of chronic inflammatory conditions in the intestine [30]. Moreover, PTPN2 initiates and orchestrates efficient immune responses against bacteria that penetrate the epithelial barrier [31]. The immune cells maintain intestinal homeostasis by removing invading bacteria and dying cells, secreting anti-inflammatory cytokines, and inducing/maintaining tolerance toward commensal bacteria and food particles. Loss of PTPN2 increases the inflammasome activity of macrophages by elevating the phosphorylation of ASC, the essential inflammasome adaptor protein, and leads to elevated IL-1β production. Thus, the loss of PTPN2 in macrophages causes more severe colitis, which may be mitigated by inhibiting IL-1β [22]. In addition, spermidine reduces inflammation by raising the expression and activity of PTPN2 in human THP-1 monocytes, which results in a reduction of STATs and p38 MAPK signaling, and IFN-γ induced expression/secretion of certain pro-inflammatory cytokines [32].

### 2.3. PTPN2 in Other Inflammatory Reactions

**Figure 2:**
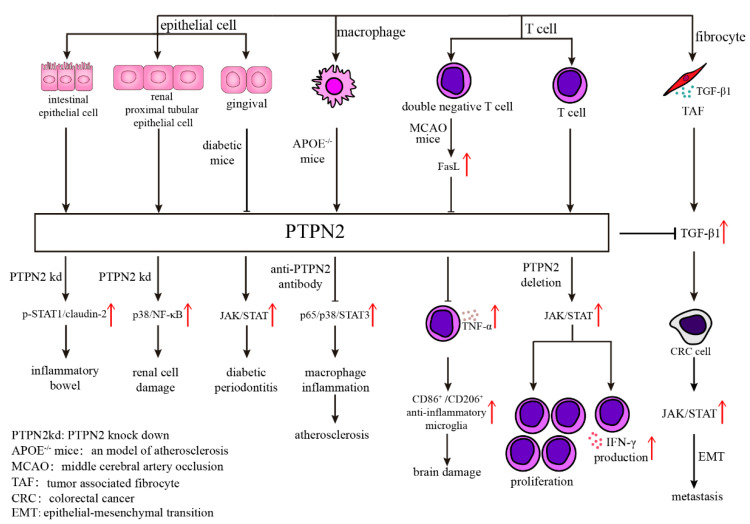
Various mechanisms of PTPN2 in different kinds of cells. Abnormal expression of PTPN2 in epithelial cells will result in many diseases through different mechanisms, such as p-STAT1/claudin-2 in inflammatory bowel, p38/NF-κB in renal cell damage, and JAK/STAT in diabetic periodontitis. The antibody of PTPN2 in APOE^−/−^ mice inhibits atherosclerosis through diminishing p65/p38/STAT3 signaling pathway. What’s more, PTPN2 influences T cell function, including cell proliferation and IFN-γ production, through JAK/STAT signaling pathway.

Atherosclerosis is the primary cause of cardiovascular disease. Systemic inflammation is an important characteristic of atherosclerosis, which is aggravated by the inflammatory factors secreted by pro-inflammatory macrophages. PTPN2 assists in inhibiting the release of inflammatory factors in macrophages via de-phosphorylating p65/p38/STAT3 in an atherosclerosis model. The results indicate that PTPN2 plays a negative role in the occurrence of atherosclerosis by inhibiting the secretion of inflammatory factors in macrophages and may be a treatment candidate for atherosclerosis (Figure 2) [33].

Neuro-vascular inflammation is characterized by the breakdown of the blood-brain barrier (BBB) and increased endothelial permeability, which leads to cerebral edema, a condition that can occur in a range of illnesses such as stroke, trauma, tumor, infection, and degenerative diseases [34,35,36,37]. Angiopoietin-1 (Ang-1) diminishes thrombin-induced breakdown of the BBB by mediating disruption of tight junctions (TJs), which are involved in tyrosine phosphorylation of Occludin (a major component of TJs). Depletion of PTPN2 eliminates Ang-1 function that promotes tyrosine de-phosphorylation of Occludin, and endothelial hyperpermeability. The results indicate that PTPN2 blockage via mediating tyrosine phosphorylation of Occludin is closely tied to maintaining BBB function and may be a novel therapeutic target for neuro-inflammatory disorders associated with BBB disruption [38]. Regarding neuroinflammation after ischemic stroke, CD3^+^CD4^−^CD8^−^ T cells (double-negative T cells; DNTs) dramatically increased in stroke patients and in a mouse model in a time-dependent manner, which exacerbates cerebral immune and inflammatory responses and ischemic brain injury via TNF-α production and the following proinflammatory microglial activation. This process involves the FasL/PTPN2/TNF-α signaling pathway, in which FasL activation promotes TNF-α production in DNTs, where PTPN2 serves as a negative regulator of FasL signaling to suppress TNF-α secretion (Figure 2) [39].

PTPN2 also contributes to the regulation of renal inflammation. In HK-2 cells (a classical model of sepsis-induced renal injury in vitro), PTPN2 reduced LPS-induced inflammatory cytokine release and cell death via modulating p38 MAPK/NF-κB signaling, which alleviated renal cell damage by playing a nephron-protective role in sepsis-induced renal injury (Figure 2) [40].

Moreover, PTPN2 plays a part in inflammation-associated metabolic disorders, such as diabetes-related diseases. Diabetic nephropathy (DN) is a chronic inflammatory kidney disease caused by diabetes. Recent studies have shown that PTPN2 exerts protective effects by ameliorating metabolic disorders and suppressing micro-inflammation via the STAT signaling pathway, which suggests PTPN2 is a potential target for the treatment of human DN [41]. In addition, the interaction between PTPN2 and JAK/STAT pathway may contribute to the development of diabetic periodontitis [42].

### 2.4. PTPN2 Regulates the Development and Redistribution of T Lymphocytes

Both the development of T lymphocytes in the thymus and the activation of mature T lymphocytes in secondary lymphoid tissues require that the lymphocytes respond adaptively to environmental signaling molecules. The T cell receptor (TCR) interaction with the MHC/antigen peptide complex, together with the CD4 and CD8 co-receptors’ interaction with the co-stimulatory molecules and cytokine receptor-mediated signals, activates the TCR signaling pathway and leads to an immune response. The lymphocyte-specific protein tyrosine kinase (Lck) and proto-oncogene tyrosine-protein kinase (Fyn) kinases, members of the Src family of non-receptor tyrosine kinases, influence T lymphocyte activation, differentiation, and tolerance [43]. Lck and Fyn are proximal signal proteins that are activated in the TCR signaling pathway.

Protein tyrosine phosphatases (PTPs) play an important role in T lymphocyte development and function. Among these, PTPN2 has different qualitative or quantitative effects on the early activation, proliferation, survival of mature T lymphocytes, differentiation of T cells, and regulation of T lymphocyte subsets. 

T cell progenitors transition through T cell differentiation through the IL-7-STAT5 axis, of which the target genes change dynamically. IL-7R modulates gene expression via the JAK-STAT pathways, and STAT5 is the primary STAT family member activated downstream of IL-7R [44]. PTPN2, a negative regulator of IL-7R-STAT signaling, contributes to the nature of STAT-mediated gene targeting in T cell differentiation. A lack of PTPN2 expression results in an abnormal interferon-response gene profile due to amplified phosphorylation of STAT family members, which leads to the deregulation of early development checkpoints and the ensuing inefficient differentiation of CD4^+^ CD8^+^ double-positive lymphocytes [45]. PTPN2 also dephosphorylates TCR-proximal kinases, such as Lck and Fyn, which results in the increase of the threshold for T cell activation, and subsequent reduction of the sensitivity to low-affinity antigens during T cell signaling [46,47,48] Generally speaking, lack of PTPN2 causes broad changes in the expression and phosphorylation of T cell expansion and survival-associated proteins, which renders cells less dependent on survival-promoting cytokines. Thus, PTPN2 deficiency leads to: (1) augment of programmed T cell expansion and survival capacity of activated T cells [11]; (2) enhancement of T cell signaling [48]; (3) promotion of CD8^+^ T cell responses after antigen cross-presentation [46]. In lymphopenia-induced proliferation (LIP), a condition that contributes to the onset of inflammatory bowel disease, rheumatoid arthritis, and type I diabetes [49], some T cells expand due to the recognition of self-antigens and/or cytokines, particularly IL-7. PTPN2 is strongly engaged in this process, with its expression elevated in naive T cells that leave the thymus to restrict homoeostatic T cell proliferation and prevent excess responses to self-antigens in the periphery [47]. Consequently, PTPN2 deficiency leads to an elevated T cell receptor-dependent response and the further development of autoimmunity. As a result, negative regulation by PTPN2 in T cells plays an important role in preventing the development of autoimmune and inflammatory disorders.

Moreover, PTPN2 engages in the regulation of T lymphocyte subsets. The PTPN2rs1893217(C) risk allele with reduced PTPN2 expression causes decreased IL-2R/pSTAT5 signaling and further reduces FOXP3 expression in activated CD4+ T cells. This leads to a decrease in CD4+ FOXP3+ T cells [50]. In addition, PTPN2 deletion in CD8+ T lymphocytes increases the production, proliferation, and cytotoxicity of a Tim-3+ terminally exhausted subset without altering the number of a Slamf6+ progenitor exhausted subset in lymphocytic choriomeningitis virus clone 13 infection [51]. Additionally, a lack of Ptpn2 expression in CD8+ T cells leads to a reduction in tissue-resident memory T cells and the proportion of memory precursor cells [52]. Although PTPN2 was originally cloned from a human T cell cDNA library, it also participates in the regulation of other immune cells. Dendritic cells (DCs), which hold a crucial position between innate and adaptive immunity, modulate immunological tolerance and immune responses. Thus, DCs play a significant role in tissue homeostasis and the prevention of autoimmune responses [53]. The lack of PTPN2 in mouse DCs altered the proportion of myeloid and lymphoid immune cells in the skin, liver, lung, and kidney [54]. In THP-1 cells, loss of PTPN2 participates in inflammation-related events by promoting IFN-γ-induced STAT signaling, and IL-6 or MCP-1 secretion [55].

On the whole, PTPN2, as a prominent regulator of inflammatory and immune/autoimmune response, is a potential target to manage in order to maintain tissue tolerance.

### 2.5. The Role of PTPN2 in Immune Cells

Since PTPN2 influences the production, differentiation, and distribution of immune cells, researchers have turned the spotlight to the role of PTPN2 in the anti-tumor immune response. 

Tumors evade the cytotoxicity of the immune response primarily by (1) avoiding immune recognition and (2) instigating an immunosuppressive TME. On the one hand, PTPN2 participates in tumor avoidance of immune recognition. PTPN2 deficiency boosts the expression of human leukocyte antigens (HLAs), which causes a reduction of immune escape by presenting more antigens [13]. Additionally, PTPN2 deficiency promotes the production and secretion of T cell effector molecules, such as TNF-α and INF-γ, which increases the likelihood of detection by T cells [56].

On the other hand, PTPN2 is involved in the induction of immunosuppressive TME. PTPN2 deletion in T cells increases proliferation through elevation of JAK/STAT signaling and INF-γ production [57,58], which encourages CD4+ Th1 cell development and activation, as well as enhanced CD8+ T cell cytotoxicity [59]. PTPN2 deletion in CD8+ T cells also boosts the generation, proliferation, and cytotoxicity of Tim-3+ terminally exhausted subpopulation without altering the Slamf6+ progenitor exhausted subpopulation, which enhances anti-tumor responses and improves tumor control [51]. Additionally, PTPN2 deletion in T cells enhances the efficacy of anti-PD-1 therapy and achieves complete tumor clearance in a murine colorectal cancer model [13,59]. PTPN2 deletion of the immune system also resulted in MC38 tumor clearance and improved PD-1 checkpoint blockade responses to B16 tumors [51]. The above findings indicate that PTPN2 deletion sensitizes cancer cells to immune checkpoint therapy.

Chimeric antigen receptor-T (CAR-T) cell therapy plays a prominent role in cancer treatment. The deletion of PTPN2 in HER-2-specific CAR-T cells activates Src family kinase LCK and STAT5 signaling, enabling CAR-T cells to be activated and homed in CXCL9/10-expressing tumors to eliminate HER-2+ breast tumors in vivo. These findings define PTPN2 as a promising target for enhancing T cell tumor infiltration and tumor cytotoxicity [56].

Moreover, PTPN2 exerts a tumor-associated immunity function in other immune cells. For example, PTPN2 deficiency consistently enhances the cytotoxicity of NK cells [60]. PTPN2 deficiency in macrophages also induces the formation of inflammasomes, which convert pro-IL-1ß and pro-IL18 into their active forms via protease caspase-1 cleavage. IL-1ß has powerful pro-inflammatory properties, whereas IL-18 promotes the induction of IFN-γ-expressing cells, recruitment of pro-inflammatory phagocytes, and differentiation of Th17 lymphocytes, Th1 lymphocytes, CD8+ cytotoxic T lymphocytes, and NK cells, all of which contribute to anti-cancer immunity [61]. 

### 2.6. The Role of PTPN2 in Tumor Cells

Manguso et al. identified PTPN2 as a cancer immunotherapy target using in vivo CRISPR library screening [13]. PTPN2 deletion increases IFN-γ-induced STAT1 phosphorylation, expression of antigen processing and/or presentation related molecules (Tap1, Tapbp, B2m, MHC-I, and MHC-II), as well as expression of some chemokines (Cxcl9, Cxcl10, Cxcl11, and Ccl5), which recruit T cell infiltration into the tumor. PTPN2 levels are upregulated in human cancers that are refractory to current immunotherapy. PTPN2 deletion in tumor cells improves immunotherapy effectiveness by boosting interferon-γ-mediated signaling and growth suppression [13]. Ptpn2-null B16 tumors contain a significantly greater number of CD8+ T cells (especially an increased fraction of activated, cytotoxic CD8+ T cells) and γδ+ T cells, but no difference in the number of CD45+ cells, NK cells, CD4+ T cells, FoxP3+ regulatory T cells, or cells in the myeloid compartment.

Many studies have corroborated the role of PTPN2 as a tumor suppressor in cancers. However, as more and more comprehensive and detailed studies have emerged, PTPN2 has been implicated in cancer-promoting effects and poor prognoses. PTPN2 negatively regulates the tyrosine phosphorylation of KRAS, which affects its plasma membrane localization and downstream signal transduction. Thus, PTPN2 knockdown significantly reduced proliferation and promoted apoptosis in KRAS-dependent cancer cells (HCT-116, PaTu8988T, and H460), but not in KRAS-independent cells [62]. Moreover, PTPN2 positively regulated mitochondrial respiration in HCT116 human colorectal cancer cells via STAT3 phosphorylation. Deletion of PTPN2 leads to an increase in the number of damaged mitochondria, which prevents increased expression of electron transport chain-related genes and decreases ATP production, cellular proliferation, and migration [63].

Based on T cell infiltration and levels of pro-inflammatory cytokines, tumors are divided into immune-cold tumors (such as breast cancer, ovarian cancer, prostate cancer, etc.) and immune-hot tumors (such as melanoma and non-small cell lung cancer) [64,65,66]. PTPN2 is considered one of the most potential immunotherapy targets for exacerbating IFN-g signaling networks and the downstream response to ICI immunotherapy. PTPN2 exerts the above-mentioned function mainly via the JAK/STAT signaling pathway in either immune-cold or immune-hot tumors [67]. Although protein kinase B (AKT), Src family kinase (SFK), MEK/ERK pathways have also been reported to interact with PTPN2 signaling, JAK/STAT is still the major signaling pathway to enhance the efficacy of ICIs in tumors [3,68,69].

### 2.7. Gastrointestinal Cancer

For stomach adenocarcinoma (STAD) and colorectal cancer (CRC), PTPN2 expression levels are correlated with cancer incidence. According to the analysis results of 715 datasets in Oncomine, PTPN2 was highly expressed in gastric cancer cell lines and GC tissues [70]. The PTPN2 expression level was significantly increased in CRC tumor tissues of all stages. Furthermore, PTPN2 expression is negatively correlated with low checkpoint molecule (e.g., PD-1 and CTLA4) expression in primary CRC [59]. Moreover, TAFs (tumor-associated fibroblasts) contribute to CRC’s metastasis via secreting TGF-1β and JAK/STAT signaling pathway in CRC, which is inhibited by PTPN2 (Figure 2) [71].

### 2.8. Breast Cancer

The biological functions of PTPN2 vary according to breast cancer subtypes. PTPN2 is associated with worse patient outcomes in Luminal A and HER2+ subtypes, whereas it does not seem to play a prognostic role in TNBC [72]. PTPN2 gene loss is a significant predictive marker of poor benefits from tamoxifen treatment and is associated with higher levels of nuclear-activated Akt in ER-positive breast cancers [73,74]. Loss of PTPN2 enhances SFK and STAT3 signaling, as well as tumorigenicity in human breast cancer cells in vitro and in vivo [75]. However, PTPN2 deletion increases STAT-1-dependent T cell recruitment, PD-L1 expression, and the resultant enhanced efficacy of anti-PD-1 in murine TNBC models. Thus, the biological function of PTPN2 in breast cancer is a double-edged sword.

### 2.9. Glioma

PTPN2 transcript level is grade-dependent and significantly increased in isocitrate dehydrogenase (IDH) wild-type and mesenchymal subtype gliomas. PTPN2 positively correlates with immune cell infiltration, including macrophages, neutrophils, and CD8+ T cells [76]. Thus, PTPN2 is an indicator of worse overall survival in patients with gliomas and glioblastomas. PTPN2 contributes to glioma progression either via inflammation cytokines interferon-γ and TNF-α, or oxidative stress [77]. TC45, a 45-kDa variant of PTPN2, has the potential to dephosphorylate Delta EGFR, which is the most common mutation of the EGFR gene and promotes glioblastoma multiforme (GBM) growth. TC45 inhibits the proliferation and anchorage-independent growth, as well as the growth of Delta EGFR-expression cells in vivo, and prolongs the survival of mice implanted with the tumor cells via the mitogen-activated protein kinase ERK2 and PI3K pathway [78].

### 2.10. Hepatocellular Carcinoma

The oxidative hepatic environment in obesity induces PTPN2 inactivation and the resultant enhanced STAT1 and STAT3 signaling, which promotes T cell recruitment and ensures non-alcoholic steatohepatitis and fibrosis, as well as hepatocellular carcinoma. Moreover, PTPN2 deletion in hepatocytes significantly accelerated chemical carcinogen-induced HCC in mice. However, only STAT3, but not STAT1 signaling, is involved in the PTPN2-associated pathogenesis of HCC [79].

### 2.11. Skin Cancer

PTPN2 is inversely correlated with the grade of squamous cell carcinoma. It also contributes to the attenuation of chemically induced skin carcinogenesis via STAT1, STAT3, STAT5, or PI3K/AKT signaling pathway. PTPN2 overexpression increased epidermal sensitivity to DMBA (7,12-dimethylbenz[a]anthracene)-induced epidermal apoptosis and decreased TPA (12-*O*-tetradecanoylphorbol-13-acetate)-mediated hyperproliferation. Moreover, PTPN2 overexpression reduced the number of tumors and presented a prolonged latency of tumor initiation during skin carcinogenesis using epidermal-specific PTPN2 overexpression mice [80,81]. In addition, PTPN2 minimizes UVB-induced epidermal cell damage by promoting apoptosis via the negative regulation of Flk-1/JNK signaling [82]. The above-mentioned findings provide evidence for the tumor suppressor function of PTPN2 in skin carcinogenesis. However, genetic inhibition of PTPN2 and ensuring amplification of JAK/STAT signaling in melanoma cells potentiated IFN-γ response and enhanced immunotherapy efficacy [67].

### 2.12. Lung Cancer

PTPN2 pathway is involved in the risk factors and survival of lung cancer. Analysis of summary data of 6 published genome-wide associated studies (GWAS) with 12,160 cases and 16,838 controls identified 11 independent SNPs of 8 genes, of which PTPN2 SNPs rs2847297 and rs2847282 may be the potential susceptible loci for the risk of getting lung cancer in the European population, particularly among ever smokers and squamous carcinoma [68]. Moreover, PTPN2 and its-linked network are associated with lung cancer metastasis [83].

### 2.13. Other Cancers

Analyzing ovarian cancer-related genes using the Cancer Genome Atlas (TCGA) and Gene Expression Ominbus (GEO) datasets identified PTPN2 as one of the protective genes for ovarian cancer. The abnormal expression of PTPN2 is highly related to ovarian cancer progression, and can be used as an independent prognostic marker of ovarian cancer [84].

PTPN2 expression was higher in laryngocarcinoma cell lines in vitro, and was upregulated in laryngocarcinoma tissues, especially in those at stage 3/4. Thus, a high level of PTPN2 is a predicted poor prognosis molecule in laryngocarcinoma patients [85]. PTPN2 is also upregulated in thyroid cancer tissues and cell lines, especially in metastatic subtypes [86]. Moreover, PTPN2 is a pro-tumor gene in MYC-driven B-cell lymphoma, and contributes to tumor proliferation by promoting G1 to S phase transition of the cell cycle [87].

## 3. Conclusions

Phosphotyrosine-based signaling is essential for regulating cell growth, development, differentiation, survival, and migration. In this review, we focus on the protein tyrosine phosphatase non-receptor type 2, encoded by the PTPN2 gene, to investigate its structure, function, and role in inflammation response and tumors. PTPN2 is a non-transmembrane protein consisting of a conserved catalytic structural domain and a non-catalytic C-terminus. Due to its different shearing actions, there are two common variants of PTPN2: PTPN2 (45 kD) and PTPN2 (48 kD). The former (PTPN2 45 kD) has a hydrophilic terminus with a dichotomous nuclear localization signal, so this isoform is mainly found in the nucleus and can be transferred to the cytoplasmic region to regulate cellular activity. The latter (PTPN2 48 kD) has a hydrophobic tail and is anchored to the endoplasmic reticulum.

As a signaling molecule, PTPN2 regulates a variety of cellular processes by dephosphorylating receptor protein tyrosine kinases, whose substrates can be EGFR, CDF1R, PDGFR, IR, or non-receptor tyrosine kinases, such as JAK, Src, and STATs family kinases. A wide range of regulatory pathways are closely linked to pathological processes in inflammatory response, immune response, and tumor development. PTPN2 has multiple mechanisms for regulating inflammation, as represented by intestinal inflammation. It regulates the occurrence, progression, and regression of inflammatory responses in multiple ways by affecting intestinal barrier function, inflammatory factors, and multiple signaling pathways within inflammatory immune cells. PTPN2 function of T cell-based immune cells is most comprehensively studied, and has various qualitative or quantitative effects on early activation, proliferation, survival, differentiation, and regulation of cell subsets, such as negative regulation of IL-7R-STAT signaling, dephosphorylation of TCR proximal kinase, and interference with T cell early development checkpoints. As for the regulation of other immune cells, such as dendritic cells, PTPN2 also plays an important role.

In tumors, PTPN2 affects tumor cells and tumor-associated cells, thereby influencing tumor progression. Although a large number of previous studies have confirmed the tumor suppressive effects of PTPN2 in human cancers, PTPN2 has some degree of pro-cancer effects and predicts a poor prognosis, as more and more comprehensive and detailed studies have emerged. We enumerated the positive or negative modulatory effects of PTPN2 in a variety of cancers, including intestinal cancers, breast cancer, glioma, hepatocellular carcinoma, skin cancer, lung cancer, ovarian cancer, laryngeal cancer, thyroid cancer, and B-cell lymphoma. Because of the different functions of PTPN2 in multiple cancers, the known literature cannot provide absolutely definite clinical treatment protocols utilizing PTPN2 as a cancer target. In addition, the prognostic differences among the different disease types obtained from their cohort studies have also mystified the multi-identity gene of PTPN2. However, this does not negate the important therapeutic target that PTPN2 becomes. For example, the deletion of PTPN2 in tumor cells enhances the efficacy of immunotherapy by enhancing interferon-γ-mediated effects on antigen presentation and growth inhibition. The pro- or oncogenic role of PTPN2 needs to be further clarified in future studies, especially for different diseases and different subtypes of the same disease, or to clarify the dominant role of PTPN2 in the presence of both. Moreover, the complex cellular and stromal environment is crucial for tumor development, and whether PTPN2 regulates cellular and stromal changes within the TME is one direction for future research.

## Figures and Tables

**Table 1:** Summary table of the 107 PTP family members. PTPs can be grouped into four families based on the sequence of amino acids in their catalytic domains. The Class I cysteine PTP family is the largest family and can be categorized into two subfamilies: classical PTPs and DSPs. The other three families are class II cysteine PTP family, class III cysteine PTP family, and asp-based PTPs [4].

	Family	Subfamily	Subgroup
PTPs	Class I cysteine PTP family	Classical PTPs	Transmembrane, receptor-like enzymes (RPTPs)
			Intracellular, nonreceptor PTPs (NRPTPs)
		VH1-like, “dual-specific” protein phosphatases (DSPs)	Specific for the mitogen-activated protein (MAP) kinase
			Atypical DSPs
			Slingshots
			PRLS
			CDC14s
			PTENs
			Myotubularins
	Class II cysteine PTP family	Low Mr phosphotyrosine protein phosphatase (LMPTP)	-
	Class III cysteine PTP family	CDC25s (CDC25A, CDC25B, CDC25C)	-
	Asp-Based PTPs	EyA	-

## Data Availability

The data that support the findings of this study are openly available in Public Databases, which are included within the article.
